# Development of continuous warning system for timely prediction of septic shock

**DOI:** 10.3389/fphys.2024.1389693

**Published:** 2024-11-20

**Authors:** Gyumin Kim, Sung Woo Lee, Su Jin Kim, Kap Su Han, Sijin Lee, Juhyun Song, Hyo Kyung Lee

**Affiliations:** ^1^ School of Industrial Management Engineering, Korea University, Seoul, Republic of Korea; ^2^ Department of Emergency Medicine, Korea University Anam Hospital, Seoul, Republic of Korea

**Keywords:** early warning system, machine learning, sepsis, septic shock, artificial intelligence, time-series, electronic health record

## Abstract

As delayed treatment of septic shock can lead to an irreversible health state, timely identification of septic shock holds immense value. While numerous approaches have been proposed to build early warning systems, these approaches primarily focus on predicting the future risk of septic shock, irrespective of its precise onset timing. Such early prediction systems without consideration of timeliness fall short in assisting clinicians in taking proactive measures. To address this limitation, we establish a timely warning system for septic shock with data-task engineering, a novel technique regarding the control of data samples and prediction targets. Leveraging machine learning techniques and the real-world electronic medical records from the MIMIC-IV (Medical Information Mart for Intensive Care) database, our system, TEW3S (Timely Early Warning System for Septic Shock), successfully predicted 94% of all shock events with one true alarm for every four false alarms and a maximum lead time of 8 hours. This approach emphasizes the often-overlooked importance of prediction timeliness and may provide a practical avenue to develop a timely warning system for acute deterioration in hospital settings, ultimately improving patient outcomes.

## 1 Introduction

Early warning of clinical deterioration can provide substantial support for clinicians by facilitating prompt identification of adverse events, allowing for proactive measures or timely interventions (Muralitharan et al., 2021). Accordingly, early warning systems hold immense potential in clinical contexts, particularly where the accurate timing of recognition or treatment is paramount. Of particular interest are sepsis and septic shock, extensively examined in early warning systems due to their elevated mortality rates and diagnostic complexity.Sepsis is defined as life-threatening organ dysfunction caused by a dysregulated host response to infection, while septic shock is defined as a subset of sepsis in which underlying circulatory and cellular metabolism abnormalities are profound enough to substantially increase mortality (Singer et al., 2016) and is characterized by hyperlactataemia and hypotension requiring vasopressor therapy (Hotchkiss et al., 2016). While early treatment can improve patient outcomes (Evans et al., 2021), delayed intervention or recurring symptoms can lead to irreversible deterioration (Kumar et al., 2006). Thus, the development of an early warning system for septic shock can play a crucial role in timely treatment and prevention of recurrence.

Recent approaches to early warning systems for septic shock mainly employ data-driven machine learning based methodologies to generate warnings (Henry et al., 2015; Lin et al., 2018; Khoshnevisan et al., 2018; Darwiche and Mukherjee, 2018; Giannini et al., 2019; Liu et al., 2019; Fagerström et al., 2019; Yee et al., 2019; Khoshnevisan and Chi, 2020; Kim et al., 2020; Mollura et al., 2020; Misra et al., 2021; Wardi et al., 2021; Agor et al., 2022), enabling personalized early prediction with high sensitivity and specificity (Muralitharan et al., 2021). Most of these early warning systems aim to screen patients who are highly likely to show septic deterioration before onset as early as possible. These screening systems can be classified into two categories based on the timing of their alarm mechanisms. The first category, which we refer to as the ‘left-aligned approach’, is centered on making predictions during the initial phase of a patient’s admission. In contrast, the second category, termed as the ‘right-aligned approach’, is designed to forecast septic shock events at a specific duration prior to their actual occurrence. Thus, the ‘left-aligned approach’ aligns cohort data to the start of each patient’s admission, while the ‘right-aligned approach’ aligns data points to the onset of events or the end of a patient’s admission. However, both systems may not be clinically applicable due to their inability to timely identify the risk, as they merely predict if patients would suffer from an adverse event in the future without providing sufficient information regarding the exact time of onset, making it difficult to preemptively prepare for timely actions.

We note that the development of timely early warning systems for clinical deterioration, such as septic shock, necessitates the incorporation of three components: (1) continuous calculation of future risk based on the patient’s health status, (2) consideration of the timely adequacy of predictions based on their located time frame, and (3) appropriate evaluation of predictive performance achieved by the system. First, the incapability of alerting continuously restricts the system to making singular predictions, falling short in meeting the requisites of timeliness. Second, in the context of continuous warning systems, the establishment of a precise interval for timely warnings serves not only to accurately gauge the system’s predictive performance but also to ensure its effective management. Lastly, given the inherent disparity between a warning system designed to capture the onset of adverse events and a screening system, standard metrics employed in previous approaches may not be able to adequately measure the performance of timely warning systems.

**FIGURE 1:**
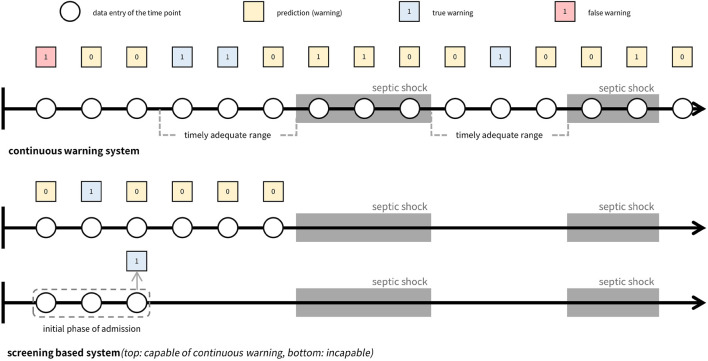
Comparison of continuous warning system versus screening system. The top figure illustrates a continuous warning system that can be managed to generate timely adequate warnings, while the figures below show two types of screening systems. Both screening systems make predictions before the event occurs and concentrates on screening whether a patient will suffer from the event in the future or not. The difference between the systems is their capability of generating continuous warnings where the first screening system is capable of generating continuous warnings while the second type is not capable.

In Table 1, prevailing studies on early warning systems for septic shock are summarized with respect to the three essential components for timeliness. To the best of the authors’ knowledge, no current frameworks satisfy all three criteria comprehensively, as most have been developed with a focus on screening rather than continuous monitoring. Although some systems leverage machine learning models capable of generating continuous warnings, such as LSTM (Long Short-Term Memory), XGBoost (Extreme Gradient Boosting), or Cox regression, and have set time windows for true warnings, these systems are still evaluated as screening tools rather than continuous warning systems. Specifically, the time windows used to define true warnings typically fall into one of three categories: the entire duration leading up to septic shock onset, the initial period post-admission, or a distant interval before the onset. As a result, despite their ability to produce continuous alerts, these systems are not optimized for issuing timely warnings. Note the difference between screening-based systems and continuous warning systems, as depicted in Figure 1.

**TABLE 1:** Summary of previous early warning systems developed for septic shock.

Author (Year)	Continuous warning	Timely range	Performance
Henry et al. (2015) ^a^	Capable but evaluated as screening system	Whole time range before the onset of target event	AUROC: 0.83, sensitivity: 0.85, specificity: 0.67, median lead time: 28.2 h
Lin et al. (2018)	Incapable	Within 12 h after admission	AUROC: 0.9411, F1 score: 0.8623, accuracy: 0.8658, recall: 0.8408, precision: 0.8849
Lin et al. (2018)	Incapable	Before 3 h from the onset of event	AUROC: 0.8647, F1 score: 0.7731, accuracy: 0.7747, recall: 0.7676, precision: 0.7931
Khoshnevisan et al. (2018)	Incapable	Within 8 h after admission	AUROC: 0.895, F1 score: 0.808, accuracy: 0.813, recall: 0.787, precision: 0.830, Dataset: EHR from Christiana Care Health System
Khoshnevisan et al. (2018)	Incapable	Before 4 h from the onset of event	AUROC: 0.943, F1 score: 0.868, accuracy: 0.875, recall: 0.826, precision: 0.915
Darwiche and Mukherjee (2018) ^a^	Incapable	Before 20 h from the onset of event	Accuracy: 0.8312, sensitivity: 0.7812, specificity: 0.8663
Giannini et al. (2019)	Capable but evaluated as screening system	Whole time range before the onset of target event	Sensitivity: 0.26, specificity: 0.98, PPV: 0.29, NPV: 0.97, median lead time: 5h 25min
Liu et al. (2019) ^a^	Capable but evaluated as screening system	Whole time range before the onset of target event	AUROC: 0.93, sensitivity: 0.88, specificity: 0.84, precision: 0.52, median early warning time: 7 h
Fagerström et al. (2019) ^a^	Capable but evaluated as screening system	Whole time range before the onset of target event	AUROC: 0.93, median hours before onset: 28.2 h
Yee et al. (2019) ^a^	Incapable	Before 24 h from the onset of the event	AUROC: 0.81, sensitivity: 0.79, specificity: 0.66, PPV: 0.46, NPV: 0.90
Khoshnevisan and Chi (2020)	Incapable	Before 48 h from the onset of event	AUROC: 0.793, F1 score: 0.737, accuracy: 0.741, recall: 0.732, precision: 0.737
Kim et al. (2020) ^b^	Incapable	At the start of ED admission (warning based on triage information)	AUROC: 0.902, AUPRC: 0.556, sensitivity: 0.706, specificity: 0.900, PPV: 0.427, NPV: 0.967
Mollura et al. (2020) ^a^	Incapable	Before 15 min from the onset of event	AUROC: 0.93, F1 score: 0.84, accuracy: 0.85, sensitivity: 0.89, specificity: 0.82, PPV: 0.80, NPV: 0.90
Misra et al. (2021)	Incapable	Within 6 h after admission	AUROC: 0.9483, sensitivity: 0.8392, specificity: 0.8814
Wardi et al. (2021) ^c^	Capable but evaluated as screening system	Before 8 h from the onset of the event	AUROC: 0.8, sensitivity: 0.85, specificity: 0.67
Agor et al. (2022) ^d^	Incapable	Before 4 h from the onset of event	AUROC: 0.9087, accuracy: 0.8312, recall: 0.7812, precision: 0.8039, specificity: 0.8663

^a^ The datasets used in these systems were from the MIMIC-II, or MIMIC-III, databases. While the MIMIC-IV, dataset may share some common cohorts with these earlier versions, the EHR, system schematics were significantly updated in MIMIC-IV, making direct comparisons between the methods of each study and our method challenging.

^b^ All performances are those from ensemble (averaging) with baseline predictors only where the target event was the onset of septic shock within 20 h after admission.

^c^ Some performances are reported just with lower bound, and specificity is reported only with a graphic, necessitating approximation.

^d^ All performances are those from logistic regression with E1 experiment result.

While prevailing research on septic shock prediction systems has not adequately addressed the importance of timeliness, other prediction systems for clinical deterioration have recognized its significance (Tomašev et al., 2019; Hyland et al., 2020). Employing various machine learning techniques, these systems were designed to trigger warnings based on real-time risk score calculations for acute kidney injury and circulatory shock. They defined prediction time windows for true warnings and optimized system performance within these windows to ensure an adequate amount of lead time before the onset of deterioration. This acknowledgment of the importance of timeliness underscores its critical role in facilitating effective disease management across various clinical contexts.

Therefore, in this study, we propose a novel approach to develop a clinically applicable early warning system that addresses all aspects of timeliness. We refer to this approach as the ‘timeliness focusing approach’, which we apply to the development of an early warning system for septic shock, named TEW3S (Timely Early Warning System for Septic Shock). Using the MIMIC-IV (Medical Information Mart for Intensive Care) database (Alistair et al., 2022), we designed TEW3S to generate continuous timely alarms every hour.

## 2 Materials and methods

### 2.1 Cohort extraction

In this study, we utilized version 2.0 of the MIMIC-IV database, a comprehensive open-source repository containing de-identified health-related data from patients who underwent intensive critical care at Beth Israel Deaconess Medical Center between 2008 and 2019 (Alistair et al., 2022). The database encompasses records of 53,569 adult ICU patients, comprising a total of 76,943 stays. A wide array of medical information, including demographic details, laboratory findings, vital signs, test results, prescriptions, pharmaceutical information, and diagnoses, were extracted from the database to construct the sequential patient data. Supplementary Tables S1, S2 provide a detailed list of the medical information employed in this research, along with the corresponding MIMIC-IV identifiers or extraction methods. Additionally, Supplementary Table S3 summarizes the average frequency of physiological signals, encompassing vital signs and laboratory results.

Given that septic shock constitutes a subset of sepsis and the diagnosis of sepsis necessitates cohorts with suspected infections (Singer et al., 2016), our study cohorts were defined as patients with suspected infections and sepsis prior to the onset of septic shock. Hence, before selecting the cohorts, we excluded those lacking variables necessary for defining sepsis or septic shock, such as systolic blood pressure (SBP), diastolic blood pressure (DBP), PaO2, FiO2, Glasgow Coma Scale (GCS), bilirubin, platelets, creatinine, and lactate.

Suspected infection was defined for admissions meeting three conditions: (1) received antibiotics, (2) blood culture tests had been taken, and (3) infection-related ICD-9 or 10 codes had been issued. Sepsis was only defined for cohorts with suspected infections, with its onset marked when the Sequential Organ Failure Assessment (SOFA) score reached or exceeded two points. Septic shock was only defined after the onset of sepsis, resulting in the exclusion of cohorts where septic shock occurred prior to sepsis. This decision is based on the assumption that timely prediction is more effective in cases where sepsis precedes septic shock, compared to cases where septic shock occurs prior to sepsis, as early intervention is more likely to have already taken place in the latter instances. Note that this approach can lead to the restriction of our research cohort to patients with nosocomial septic shock, and as such, our predictive model may not be applicable to cases of non-nosocomial septic shock. The onset of septic shock was determined when the lactate level equaled or exceeded 2 mmol/L and vasopressor therapy was administered, given that the definition of septic shock includes hyperlactataemia and vasopressor therapy (Hotchkiss et al., 2016).

### 2.2 Data refinement

We refined the data through several steps, including unit unification, outlier removal, adjustment of time errors, and correction of variable-specific errors. Initially, unit unification was applied to variables with measurements in different units, such as height, weight, temperature, vasopressors, and fluids. We consulted with professional clinicians to establish outlier criteria, drawing on the guidelines from (Hyland et al., 2020), as detailed in Supplementary Table S4. This ensured alignment with both theoretical considerations and practical feasibility in clinical settings. For instance, heart rate values were accepted within the range of 0–300 beats per minute, as values below 0 are theoretically impossible, and values above 300 are extremely rare in clinical practice. Entries falling outside these criteria were identified as errors and subsequently removed.

Time errors, defined as data entries assigned to a patient sequence with timestamps incongruent with the sequence, were adjusted. Entries recorded more than 2 days before admission or 2 days after discharge were deleted. For variables with specific timestamps, such as lab values, entries recorded outside the interval between ICU admission and discharge were excluded. Conversely, for variables recorded continuously, such as pharmaceutical variables and ventilator data, entries with start and end times outside the admission-to-discharge interval were omitted.

Additionally, errors specific to GCS and urine output were addressed. GCS comprises three component variables (eye, motor, and verbal), and its calculation relies on the summation of these components, necessitating consistency in the recorded timing of each variable. For every timestamp of the GCS components, we assumed all GCS information were recorded but some random missing entries could occur. To handle missing values, we employed a forward-and-backward imputation strategy. Urine output calculations involve unique variables, including irrigant in and out values. To accurately measure urine output at specific time points, we subtracted cumulative irrigant in amounts from cumulative irrigant out amounts. For irrigant out values immediately followed by irrigant in values, we recorded the cumulative sum of irrigant out values minus the cumulative sum of irrigant in values, retaining these records for further preprocessing. In cases where no irrigant in values preceded irrigant out values, we assigned the irrigant in value as 0.

### 2.3 Sequential merging and resampling

As some data entries were distributed across distinct datasets using different identifiers despite representing the same variable, merging the data into a sequential representation was necessary. We consolidated data entries in the MIMIC-IV datasets according to their respective variables. For entries categorized as vital signs, lab results, height, and weight, all values and corresponding timestamps were collated into a unified sequential timeline for each variable. Pharmaceutical instances were aggregated based on shared timestamps, while administration rates were listed individually. Age at admission and gender were also incorporated into the sequential data. Variables used to define sepsis and septic shock shared identical timestamps and were imputed accordingly based on variable-specific schemes. We adhered to predefined definitions for sepsis and septic shock, excluding cohorts without sepsis and those where sepsis occurred after the onset of septic shock. Subsequently, we performed data resampling, discretizing the concatenated data into predefined time intervals by aggregating or averaging variable values. A 1-h interval was chosen, considering both the dynamic nature of septic shock and the practical frequency of warnings in clinical settings.

For feature engineering, summary statistics were computed within each time interval, including the mean, median, maximum, and minimum values, with the mean serving as the representative value. Additionally, slope features were generated by calculating the difference between values at current and past time points (one, three, and 5 hours prior). These features capture temporal dynamics within and across intervals, facilitating the predictive model’s learning process. Features were not derived for unsuitable variables such as age, drug-related items, and ventilator data. In cases where no feature values were available for a given interval, different imputation methods were applied based on the nature of the data. Lab-related features were imputed using backward and forward filling, while vital signs (excluding GCS), height, and weight were linearly interpolated. This choice of imputation methods reflects the typical frequency with which these variables are recorded in clinical practice (See Supplementary Table S3). Lab measurements are usually taken less frequently and sporadically, so forward and backward filling ensures that the last known value is carried forward until a new measurement is available, preserving temporal continuity. In contrast, vital signs are monitored more frequently, allowing for the use of linear interpolation to estimate values between measurements, which assumes a more gradual and consistent change over time. If a variable was not recorded at all across the cohort, all values for that variable were imputed as 0. To distinguish true zero feature values from imputed ones, we appended presence features indicating whether values were filled by imputation (0) or not (1). This approach accounts for the uncertainty of feature values during prediction generation, as proposed in warning systems for acute kidney injury (Tomašev et al., 2019). Finally, we defined sepsis and septic shock and excluded cohorts using the same procedures as in the sequential merging process. The resulting resampled dataset comprised 11,780 stays, of which 4,369 exhibited septic shock. We partitioned the dataset into training (70%), validation (10%), calibration (10%), and test (10%) sets.

### 2.4 Timeliness focusing approach via data-task engineering

The ultimate aim of our approach was to demonstrate a clinically applicable early warning system via successful integration of timeliness within the development course. In pursuit of such a goal, we introduce a timeliness focusing approach which encompasses three main considerations.

First, to ensure the clinical relevance of our system, we evaluated predictive performance from multiple perspectives. We assessed performance not only on all instances of shock onset but also specifically on the first occurrences of shock, which may hold greater clinical significance. Compared to recurring septic shocks, the first onset of septic shock may be of more clinical value as clinicians may not have been aware of the patient’s deteriorating health status. Furthermore, we analyzed performance variations by adjusting the definition of timely warnings through what we termed the ‘evaluation window’, exploring different time points relative to shock onset to accommodate varying clinical needs. Relative to the septic shock onset time denoted as t = 0, the earliest time point of the evaluation window was defined as t = −8 and the latest time point as t = 0. Varying time points between t = −8 and t = 0 were employed to assess the robustness of our system against the varying needs of specific clinical application contexts.

Second, in addition to standard metrics used in screening systems or machine learning models, we introduced two metrics to measure timeliness: Target Event Recall (TER) and True Alarm Rate (TAR). TER measures the proportion of events warned by timely alarms, while TAR quantifies the fraction of timely warnings among both false and timely warnings. Timely warnings are defined as those occurring within the evaluation window, while false warnings exclude those generated during prolonged septic shock events. Although alarms occurring during prolonged shock events fall outside the evaluation window, they remain critical indicators of ongoing elevated risk and should not be classified as false alarms. Furthermore, we utilized modified versions of TER and TAR, termed ‘TER stay’ and ‘TAR stay’, respectively. These metrics provide an average assessment of TER and TAR specifically for stays with septic shock.

**FIGURE 2:**
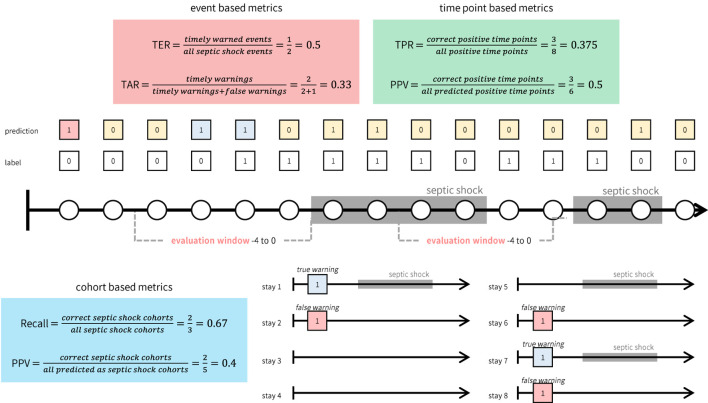
Illustrative example of how event, time point, and cohort based metrics are calculated. While event based metrics (red box) and time point based metrics (green box) are computed based on the continuous warnings generated by the system, cohort based metrics (blue box) can only be computed for each cohort.

As defined, TER and TAR are calculated for individual septic shock events, which may differ from evaluation metrics commonly used in conventional machine learning classification tasks (e.g., True Positive Rate) or those in prevailing screening systems for septic shock. To distinguish these metrics, we term the metrics introduced in this study (TER and TAR) as ‘event-based metrics’. In contrast, standard machine learning task metrics evaluate predictions at each time point, while screening system evaluation metrics are computed for each cohort (e.g., the proportion of cohorts adequately predicted). Hence, we classify the conventional evaluation metrics from machine learning tasks as ‘time point-based metrics’ and those from screening systems as ‘cohort-based metrics’. Figure 2 illustrates the differences between these three types of metrics.

Third, to optimize the timeliness of our early warning system, we investigated the impact of various factors on prediction timeliness. These factors encompassed model architecture, deliberate data provision, and the utilization of calibration and oversampling techniques. Our primary focus was placed on data provision, which involves selecting data samples for training and designing prediction tasks with different time windows (prediction window). We termed this approach ‘data-task engineering’, akin to feature engineering, as it aims to optimize predictive performance by manipulating the relationship between input data and prediction targets. This approach distinguishes itself from traditional machine learning-based early warning systems, where timeliness is often overlooked. Even when considered, the typical methodology involves training models with fixed prediction windows and including all possible data samples in the training set. We hypothesized that each data entry possesses distinct characteristics depending on its relative timing to the onset of target events. Thus, systematic inclusion of data samples can guide the model to learn the intended relationship between input data and target events.

**FIGURE 3:**
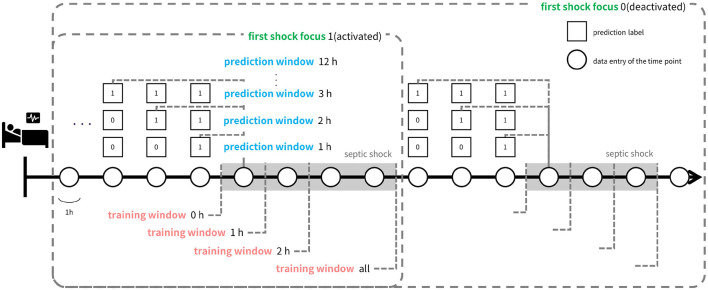
Illustration of data-task engineering approach. Data-task engineering involves three schemes: Prediction window (blue text) alters data labels by labeling the time points within prediction window prior to septic shock onset as positive labels. Training window (red text) determines how much time points after shock onset to include in the train set and first shock focus (green text) controls whether to include data points after the first shock onset in the train set. Thus, prediction window is related to the task of the learning objective while training window and first shock focus controls train set data.

As depicted in Figure 3, data-task engineering encompasses three distinct schemes, each tailored to capture specific correlations between the samples and the prediction tasks. The first scheme involves manipulating the prediction window, adjusting the timeframe from 1 hour to 12 h. This variation alters the nature of the tasks learned by the model. The second scheme centers on restricting the use of data after the onset of septic shock. Specifically, we confine the training data to a window spanning from zero to 2 hours post-onset, termed the ‘training window’. Additionally, we consider utilizing all training data samples post-shock onset, labeled as training window ‘all’. Lastly, in the third scheme, we experiment with retaining only the data entries around the initial occurrence of septic shock, referred to as ‘first shock focus’. Through data-task engineering, we aim to further refine the model’s learned function, thus enhancing predictive performance beyond conventional approaches.

Focusing on the importance of timeliness, we devised a comprehensive modeling and validation process. Initially, we trained and validated the system by exploring various combinations of model architecture, data-task engineering schemes, and auxiliary techniques such as oversampling and calibration. We assessed the predictive performance of each combination, aiming to exceed a clinically applicable threshold. This threshold was meticulously determined in consultation with clinical experts and was defined as a TER of 0.9 and a TAR of 0.2 when predicting all shocks within the evaluation window of −8 to 0. Throughout the training and validation process, our primary objective was to improve TER while maintaining a TAR of 0.2. Once combinations surpassing the threshold were identified, we employed an ensemble approach to consolidate these into the final early warning system, TEW3S. This rigorous approach ensured that our system met the clinical requirements for timely detection of septic shock.

### 2.5 Predictive modeling for TEW3S

TEW3S was developed using supervised machine learning models, including CatBoost (Hancock and Khoshgoftaar, 2020), LightGBM (Ke et al., 2017), XGBoost (Chen and Guestrin, 2016), Random Forest (Breiman, 2001), Logistic Regression (Wright, 1995), Decision Tree (CART) (Lewis, 2000), and Multinomial Naive Bayes (Webb et al., 2010). Our predictive model was designed to generate timely predictions every hour, leveraging current-hour data entries that encompassed not only the mean values but also temporal variability features within and across time steps which were derived through feature engineering, along with presence features to enhance model performance. Throughout the development process of TEW3S, auxiliary techniques such as oversampling and calibration were employed. Oversampling techniques like SMOTE (Synthetic Minority Over-sampling Technique) (Chawla et al., 2002) and ADASYN (Adaptive Synthetic Sampling) (He et al., 2008) were utilized to balance the class distribution, while isotonic and sigmoid regression were used for calibration of resultant risk score of prediction models. The hyperparameters of each model were set as Supplementary Table S5.

In assessing the timeliness of model predictions, we also calculated time point-based metrics such as the area under the precision-recall curve (AUPRC). AUPRC aided in selecting candidate settings during training and validation, complementing timeliness metrics by capturing the density of warnings within prediction windows. High AUPRC values, coupled with high timeliness, indicated a high true alarm rate, underscoring the importance of incorporating AUPRC in the training and validation process.

The overall training and validation process for TEW3S comprised three main steps. Initially, we trained and evaluated various supervised machine learning models with several training datasets engineered by data-task engineering scheme combinations, selecting those surpassing clinically applicable thresholds of TER and TAR. We further refined our selection based on time point-based AUPRC metric by identifying combinations with AUPRC values that exceeded the average of selected combinations. Note that these combinations consisted of which machine learning model and data-task engineering schemes to use. Subsequently, we applied auxiliary oversampling and calibration techniques to enhance either TER or TAR and diversified the pool of training settings for constructing ensemble models. This phase yielded 31 distinct training settings that met the clinically applicable criteria for an early warning system, defined as TER 0.9 and TAR 0.2. In the final phase, both soft and hard voting ensembles were constructed using the clinically applicable settings, with the hard voting ensemble outperforming the soft voting ensemble. Thus, we identified the hard voting ensemble as our ultimate choice for TEW3S, an early warning system tailored for septic shock. We implemented this predictive modeling process using Python 3.9 and relevant libraries, including Numpy, Pandas, Matplotlib, and Sklearn.

### 2.6 Calculation of misalignment between event versus cohort and time point-based metrics

To accurately gauge the predictive timeliness of TEW3S, we relied on event-based metrics, namely, TER and TAR. These metrics offer unique advantages over conventional types of metrics, including both cohort-based and time point-based ones, not only in terms of their intrinsic meanings but also based on the results of numerical experiments. This distinction becomes evident when examining the discrepancy between event-based metrics and other metrics, which is calculated as follows:

1. Initially, we determined the proportion of training settings, comprising various combinations of models and three data-task engineering schemes, that exhibited clinically applicable timeliness (i.e., TER 0.9 and TAR 0.2 within the evaluation window of −8 to 0). We denote this proportion as ‘p’ and the set of settings meeting these criteria as ‘C’. Note that in this context, settings involving oversampling and calibration techniques were excluded, as these auxiliary techniques were only applied to a subset of the training settings.

2. For the cohort and time point-based metrics which include measures related to both event sensitivity and alarm precision, we calculated the one-p percentile of these metrics.

3. Subsequently, for each cohort- and time point-based metrics, we computed the proportion of settings within set ‘C’ that failed to achieve the one-p percentile of the respective metric. This proportion represents the level of discrepancy observed in settings that demonstrated high timeliness but attained lower-ranked performance in other metrics.

This calculated discrepancy proportion underscores the indispensable role of TER and TAR in evaluating predictive timeliness effectively.

## 3 Results

### 3.1 Predictive performance of TEW3S

**FIGURE 4:**
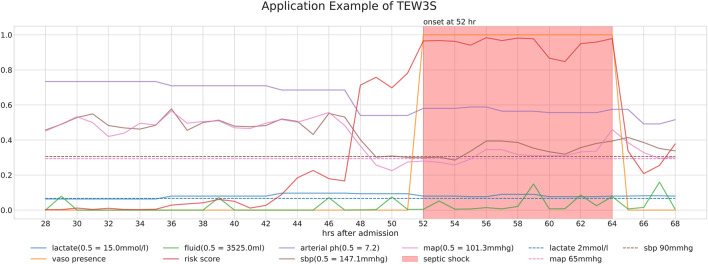
Detailed case study of a septic shock patient from our study cohorts, illustrating the sequential information relevant to their condition. The figure displays the patient’s risk score, derived through our timeliness-focused approach, alongside various physiological values and intervention-related variables.

Figure 4 provides a detailed case study of a septic shock patient from our study cohorts, illustrating the sequential information pertinent to their condition. It presents the patient’s risk score, derived through our timeliness focusing approach, alongside various physiological values and intervention-related variables. Note that the risk score is generated from a model utilized within the TEW3S ensemble.

The temporal changes in these variables and their correlation with the risk score yield insightful observations. During periods of low risk scores, most physiological variables remain stable, except for lactate levels. However, around the 44-h mark, a slight increase in the risk score precedes a subsequent drop in both systolic and diastolic blood pressure (SBP and DBP) approximately 5 hours later. This temporal relationship suggests our approach’s potential to predict future events. Subsequently, around the 48-h mark, a significant spike in the risk score coincides with a sharp decline in both SBP and MAP, falling below critical clinical thresholds of 90 mmHg and 65 mmHg, respectively, further validating the physiological plausibility of the risk score’s increase. Interestingly, despite poor physiological signs after the 44-h mark, there are instances where the risk score decreases, typically following fluid administration. However, the risk score remains significantly elevated after the onset of septic shock at the 52-h mark, persisting until the end of the observation period, even as other physiological variables return to pre-septic shock levels. This persistence suggests the model’s ability to recognize the heightened risk associated with the septic shock state.

Ultimately, our predictive model aims to generate alerts based on the risk score, starting 8 h before the onset of septic shock. The risk score notably begins to rise distinctly from the 44-h mark, precisely 8 h prior to the event, underscoring its predictive capability. Therefore, the TEW3S, resulting from an ensemble of such risk score-based models, demonstrates excellent performance in reflecting both current and future physiological states and the risk of septic shock, offering valuable insights for clinical practitioners. Given the exemplary predictive performance demonstrated in this single case, we further evaluate the predictive accuracy of the hard ensemble-based model TEW3S across all patient stays. By utilizing a hard voting ensemble of models that surpass the predefined threshold, TEW3S achieved a strong performance of TER of 0.9403 and TAR of 0.2018 when predicting all shocks within the evaluation window of −8 to 0. The detailed predictive performance of the early warning system is presented in Table 2. In summary, TEW3S accurately identified 94.0% of septic shock onsets and 93.1% of first septic shock onsets within an 8-h window, with an average of one true alarm for every four false alarms. Additionally, TAR stay, representing the average true alarm rate among septic shock cohorts, reached 0.43 for predicting all shocks and 0.77 for predicting the first shock in the evaluation window of −8 to 0.

**TABLE 2:** Predictive performances of TEW3S in evaluation window –8 to 0.

Evaluation metric	All shock	First shock
TER	0.9403	0.9314
TAR	0.2018	0.1784
TER Stay	0.9314^a^	0.9347
TAR Stay	0.4305	0.7717

^a^ TER, stay of all shock prediction always equals to TER, of the first shock prediction.

To assess TEW3S’s effectiveness in clinical settings, we analyzed the number of septic shock events predicted by timely alarms before clinicians initiated interventions (i.e., treatments for septic shock). We defined the initiation of septic shock intervention as the time point of vasopressor and fluid co-administration. Alarms triggered prior to this intervention start time were considered timely. Remarkably, 49% of septic shock events were anticipated by these timely alarms, implying almost half of septic shock events were identified through timely alarms preceding clinicians’ interventions, demonstrating the practical utility of TEW3S in clinical practice.

Additionally, we analyzed TER within different evaluation windows, ranging from −8 to −4 as the earliest time point and −2 to 0 as the latest. The sensitivity analysis results presented in Table 3 illustrate that TEW3S successfully identified over 75% of septic shock events 2 hours prior to onset in the evaluation window starting from −8. Even when considering alarms only within 4 h prior to onset, 91.7% of all septic shock events were accurately predicted in advance. Notably, nearly 70% of septic shock events were timely warned by TEW3S even in the most restrictive evaluation window of −4 to −2, highlighting its robustness across various scenarios.

**TABLE 3:** TER variation in various evaluation windows.

Evaluation Window	−8 to 0	−8 to −1	−8 to −2
TER	0.9403	0.8230	0.7537
Evaluation Window	−7 to 0	−7 to −1	−7 to −2
TER	0.9382	0.8166	0.7452
Evaluation Window	−6 to 0	−6 to −1	−6 to −2
TER	0.9307	0.8049	0.7324
Evaluation Window	−5 to 0	−5 to −1	−5 to −2
TER	0.9254	0.7953	0.7175
Evaluation Window	−4 to 0	−4 to −1	−4 to −2
TER	0.9168	0.7836	0.6962

In comparison to existing literature, we further assessed TEW3S’s predictive performance using cohort-based metrics, including sensitivity, specificity, precision, accuracy, and the F1 score. Note that as TEW3S was constructed using a hard-voting ensemble, AUROC could not be utilized. Given the focus on timeliness, our main evaluation metrics are event-based metrics (TER and TAR) as they are most appropriate for continuous warning systems. Conventional metrics were utilized for comparison purposes only, as they cannot fully capture the performance of continuous warning systems. In evaluating these metrics, we considered only the initial warning when labeling the entire cohort as positive. Consequently, TEW3S demonstrated a sensitivity of 0.9634, specificity of 0.4818, precision of 0.5230, accuracy of 0.6604, and an F1 score of 0.6779. When compared to previous research (Henry et al., 2015; Liu et al., 2019), which reported sensitivities of 0.85 and 0.88, and specificities of 0.67 and 0.84, respectively, TEW3S’s predictive performance aligned closely with these prior approaches. It is worth noting that although TEW3S was primarily designed for superior timeliness rather than optimal screening performance, its effectiveness was comparable to these established models. This suggests that our proposed methodology allows for the development of an early warning system proficient not only in generating timely continuous warnings but also in effectively screening high-risk cohorts.

We further carried out failure case analysis, examining both false alarms and instances where timely alarms were absent. Several rational causes of failures were identified. First, for false alarms, we found that 95% of false alarms were associated with vasopressor, fluid administration, or mechanical ventilation within a 3-h interval. This suggests that most false alarms adequately reflected real patient risk, but subsequent septic shock onsets could have been prevented due to timely treatment by clinicians. Second, for the false negative cases, we observed that 13% of stays without timely warnings experienced a rapid onset of septic shock within 12 h after admission. This indicates that TEW3S may not have had sufficient time to generate timely predictions in these instances. Third, we compared the average values of clinical variables for each failure case: those with false negative cases versus false positive cases (refer to Table 4). The comparison revealed that false warning cases exhibited a worse health state on average than no warning cases. This difference was particularly notable in lactate levels, where the average lactate value for no warning cases was 1.36, whereas for false warning cases, it was 2.42. The proportion of false negative stays with lactate value below 2, 1.5, and 1.1 were 85.7%, 73.5%, and 26.5%, respectively, implying the predictive capability of TEW3S heavily relies on lactate value. Lastly, we extended the timely adequate ranges used in our evaluation criteria. Given that patient risk of septic shock may extend beyond the current 8-h window prior to onset, we evaluated warnings within 24, 48, and 96 h before septic shock onset. This adjustment led to a decline in the ratios of false negatives and false alarms from 4.2% to 42.4%–2.3% and 36.8%, 2.0% and 33.2%, and 1.6% and 30.0%, respectively. Notably, when considering all warnings prior to septic shock as true positives, consistent with the evaluation criteria of previous early warning systems, the proportions of false negatives and false positives dropped to 1.3% and 24.9%, respectively. This finding underscores the importance of incorporating timeliness metrics into the evaluation process and conducting a comprehensive review of the model’s predictive capabilities. In conclusion, the majority of failure cases of TEW3S may be attributed to the mitigation of risk due to timely treatment, the intractability of temporal relationships due to insufficient time before septic shock onsets, and the evaluation criteria that accepts alarms only within 8 h window prior to septic shock onset.

**TABLE 4:** Clinical variable level comparison between false negative cases and false positive cases.

Variables	False negative	False positive
MAP (Mean Arterial Pressure, mmhg)	76.75	76.41
Lactate (mmol/l)	1.36	2.42
Arterial pH	7.40	7.37
GCS (Glasgow Coma Scale)	9.98	9.33
Creatinine (mg/dL)	1.56	1.80
Bilirubin (mg/dL)	2.28	3.54
Platelets (K/uL)	217.67	194.41
SOFA (Sequential Organ Failure Assessment)	7.29	8.01

### 3.2 Misalignment of cohort, time point, and event-based metrics

As aforementioned, disparities can exist between event-based timeliness measures and time point or cohort-based metrics due to their inherent differences, emphasizing the importance of selecting adequate evaluation metrics. To explore this incongruity across various training settings, we conducted a range of analyses.

During the initial phase of the training and validation process, we observed an intriguing pattern: training settings demonstrating clinically applicable prediction timeliness did not necessarily yield commendable performances when assessed using time point or cohort-based metrics. This observation is summarized in Table 5, which outlines the proportion of training settings exhibiting this discrepancy. The proportion was calculated as the ratio of settings in which time point or cohort-based performance ranked lower than the percentile corresponding to clinically acceptable prediction timeliness. This analysis revealed that 70% of clinically applicable settings would not be retained if time point-based metrics or cohort-based metrics were the sole criteria for evaluation. This incongruity was particularly pronounced when considering cohort-based metrics, accounting for 100% of clinically applicable settings. Moreover, the maximum event-based metric value achievable among the misaligned settings, indicated by maximum TER or TAR, underscores the potential pitfalls associated with evaluating early warning systems solely based on time point-based metrics or cohort-based metrics.

**TABLE 5:** Misalignment proportions of conventional metrics.

Type of metric	Metric	Proportion of discrepancy	Max TER	Max TAR
Cohort Based	AUROC	1	0.92	0.21
Cohort Based	F1-Score	0.91	0.92	0.21
Time Point Based	AUPRC	0.70	0.91	0.21
Time Point Based	F1-Score	0.70	0.91	0.21

**FIGURE 5:**
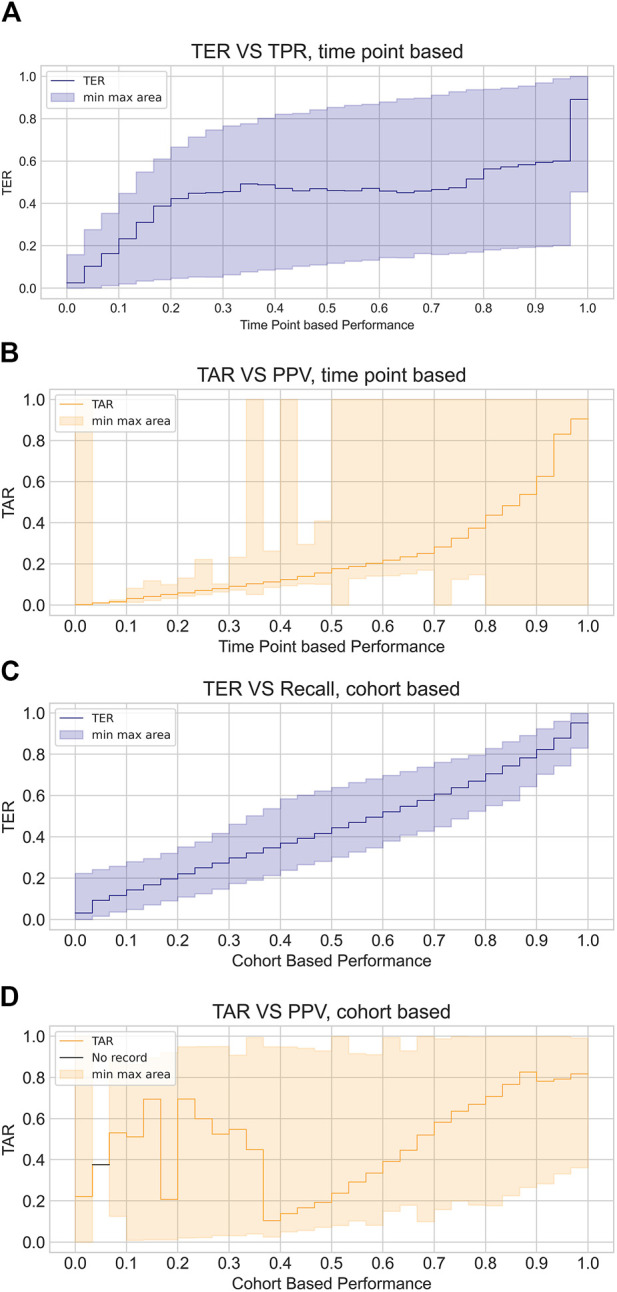
Visualized discrepancy between event based metrics and customary metrics. The plots illustrate the average event based performance corresponding to each binned intervals of time point based measures or cohort based measures, where plot **(A)** and **(B)** demonstrate TER and TAR variation with respect to time point based metrics while plot **(C)** and **(D)** show those in relation to cohort based metrics. TER is depicted in blue line and shaded area while TAR is depicted with orange line and shaded area. Line indicates average TER and TAR of each bins and shaded area shows maximum and minimum values of TER and TAR of the corresponding bins.

To further illustrate the misalignment between timeliness measured by event-based metrics and performance measured by cohort or time point-based metrics, we visualized the correlation of event-based metrics with the other two metrics, as depicted in Figure 5. From the variation of the mean TER in relation to the time point metric, we observed an almost flat or even declining trend in the middle bins, while the mean TAR in relation to the cohort-based metric demonstrated fluctuations at positive predictive values (PPV) lower than 0.4. Additionally, the shaded areas within the figures, indicating the minimum and maximum values of the corresponding TER or TAR, demonstrated a large dispersion of TER and TAR for each binned metric. Overall, the disparity between event-based metrics and other customary metrics was substantial and exhibited considerable variations.

### 3.3 Variation of prediction timeliness by data-task engineering

Based on several hypotheses regarding the impact of data-task engineering schemes and auxiliary techniques on prediction timeliness, we systematically integrated these factors into the system development process. Initially, we conjectured that factors leading to an increase in positive samples would elevate TER but decrease TAR, given their influence on augmenting the probability of positive instances within the model input distribution. Furthermore, we anticipated that data-task engineering schemes could enhance prediction timeliness while potentially introducing a trade-off between TER and TAR. For example, expanding the prediction window could broaden the model’s foresight, potentially leading to heightened overall risk assessment before shock onset. Similarly, extending the training window to include samples during septic shock prolongation might enable the model to discern physiological cues indicative of critical health states, but this could also induce overreliance on these cues. Additionally, training the model using information encompassing the dynamics around every septic shock event might render the model sensitive to predicting all septic shock onsets but less so to first shock events. These scenarios could result in higher TER but reduced TAR, while the last data engineering scheme can also provoke a trade-off of system performance on early prediction of all shock onsets versus initial onsets.

**FIGURE 6:**
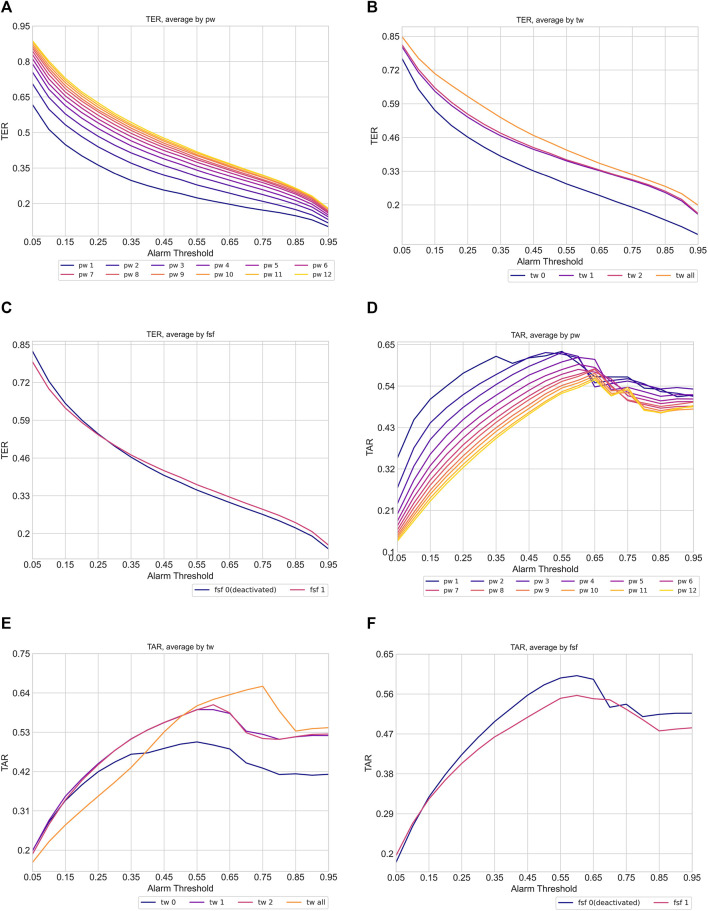
Variation of timeliness averaged by data task engineering schemes (pw: prediction window, tw: training window, fsf: first shock focus). The plot **(A)**, **(B)** and **(C)** show TER variation while plot **(D)**, **(E)**, and **(F)** indicate TAR variation. Each line in the plots indicates the average TER and TAR of corresponding data task engineering scheme configuration. The color of the line becomes brighter when corresponding scheme configuration increases.

To numerically validate these hypotheses, we computed the average TER and TAR of each data-task engineering scheme, focusing on the prediction of all shocks within the evaluation window of −8 to 0. Figure 6 presents the average TER (6a, 6b, 5c) and TAR (6d, 6e, 6f) variations along the risk threshold for each setting of the prediction window, training window, and restrictive data usage around septic shock, respectively. The visualized results support our hypotheses regarding the impact of data-task engineering schemes on TER and TAR. Overall, the plots depict a tendency where wider prediction windows and training windows, as well as using all septic shock events as training samples, tend to raise TER but decrease TAR. Moreover, the TAR variation averaged by prediction window peaked at higher thresholds as the corresponding prediction window increased, aligning with the conjecture that widening prediction windows would lead to an increase in risk scores before septic shock onset. Lastly, restricting the training set to the data entries around the first shock onset only enhanced system performances. These results suggest the existence of a trade-off for each data engineering scheme, emphasizing the need for a deliberate exploration of these schemes to achieve an optimal-performing system.

Furthermore, to demonstrate the necessity of data-task engineering, we investigated training settings that achieved high timeliness (TER 0.9 and TAR 0.2 in the evaluation window −8 to 0) using conventional early warning system development approaches. In standard development approaches without considering data-task engineering, the most commonly utilized settings would involve employing the same prediction window as the evaluation window (prediction window of eight when evaluating −8 to 0), using all data samples as the training set including those during shock duration (training window ‘all’) or not using at all (training window 0), and not differentiating between first and recurring septic shock events (first shock focus 0). Notably, there were no training settings that achieved high timeliness with conventional approaches. Even when the TER criterion was reduced to 0.85, only 1.9% of the settings comprised standard schemes. These results underscore the substantial enhancement in timeliness achieved by employing data-task engineering schemes, which were scarcely employed in previous approaches.

## 4 Discussion

In this study, we developed TEW3S, a continuous early warning system designed to timely identify septic shock by utilizing various machine learning techniques and carefully selecting training samples from the MIMIC-IV dataset. TEW3S successfully predicted 94.1% of all septic shock onsets and 93.1% of first septic shock onsets, providing a lead time of up to 8 hours at a ratio of four false warnings for every true warning. Notably, TEW3S demonstrated a high predictive sensitivity even within highly restricted windows of early warnings, managing to predict more than 75% of septic shock events 2 hours in advance and 91% of septic shock events within a 4-h window. The strong performance of TEW3S under the constraints of a timely adequate range emphasizes the effectiveness of our development approach in constructing a clinically applicable septic shock early warning system. Furthermore, despite TEW3S not originally being designed for screening high-risk patients, it achieved comparable results to previous research studies in this regard. While our system showed significant sensitivity in anticipating septic shock events, a notable number of false warnings were generated. However, many of these false alarms were associated with the initiation of interventions such as the administration of vasopressors and fluids, indicating an existing risk at the time of the alarm.

Our study introduced a novel approach focusing on timeliness in early warning system development by incorporating data-task engineering schemes and novel metrics for timeliness assessment. Analyses of timeliness metrics and the impact of data-task engineering on timeliness emphasized the importance of precise metrics for measuring the timeliness of such systems. Therefore, future efforts in developing timely early warning systems should consider data-task engineering schemes and appropriate timeliness metrics as essential components.

The primary limitations of our study stem from the architecture of the TEW3S prediction models and the absence of external validation. Although we employed a diverse array of machine learning models for prediction, we did not explore deep learning models, potentially overlooking architectures that could enhance predictive performance. Furthermore, our system was solely validated using the MIMIC-IV dataset, lacking validation on external databases which is crucial for ensuring the generalizability of our system. Additionally, as this system utilized an ensemble approach to maximize predictive performance, implementing the model in clinical practice may be burdensome. However, it is important to note that our study’s primary focus was to propose an approach for constructing a timely early warning system by emphasizing the impact of data-task engineering schemes on timeliness. Future research endeavors could delve deeper into optimizing model architectures specifically geared towards maximizing timeliness, and validate such architectures on external datasets to ensure their robustness and generalizability. Additionally, our analysis of TEW3S failure cases highlighted the association of interventions with false alarms. This suggests potential areas for future research, such as mitigating false alarms by considering intervention information. For instance, one avenue could involve suppressing alarms triggered by moderate risk levels immediately following interventions, thereby refining the system’s predictive accuracy.

Despite these limitations, our study remains novel as the first successful approach to building a timely early warning system by implementing prerequisites for timeliness and introducing data-task engineering methods. Our comprehensive analysis of timeliness sheds light on its unique characteristics compared to other types of performance metrics, highlighting the relationship between timeliness and data and task manipulation. Based on these promising results, we believe that our approach holds the potential to become a clinically applicable method for addressing acute deterioration in hospitals, potentially becoming routine clinical practice.

## Data Availability

Publicly available datasets were analyzed in this study. This data can be found here: https://physionet.org/content/mimiciv/2.0/.

## References

[B1] Agor J. K. Li R. Özaltın O. Y. (2022). Septic shock prediction and knowledge discovery through temporal pattern mining. Artif. Intell. Med. 132, 102406. 10.1016/j.artmed.2022.102406 36207079

[B3] Breiman L. (2001). Random forests. Mach. Learn. 45, 5–32. 10.1023/a:1010933404324

[B4] Chawla N. V. Bowyer K. W. Hall L. O. Kegelmeyer W. P. (2002). Smote: synthetic minority over-sampling technique. J. Artif. Intell. Res. 16, 321–357. 10.1613/jair.953

[B5] Chen T. Guestrin C. (2016). “Xgboost: a scalable tree boosting system,” in Proceedings of the 22nd acm sigkdd international conference on knowledge discovery and data mining, 785–794.

[B6] Darwiche A. Mukherjee S. (2018). “Machine learning methods for septic shock prediction,” in Proceedings of the 2018 international conference on artificial intelligence and virtual reality, 104–110.

[B2] [Dataset] Alistair J. Lucas B. Tom P. Steven H. Anthony C. L. Mark R.(2022). Mimic-iv. 10.13026/7vcr-e114

[B7] Evans L. Rhodes A. Alhazzani W. Antonelli M. Coopersmith C. M. French C. (2021). Surviving sepsis campaign: international guidelines for management of sepsis and septic shock 2021. Crit. care Med. 49, e1063–e1143. 10.1097/CCM.0000000000005337 34605781

[B8] Fagerström J. Bång M. Wilhelms D. Chew M. S. (2019). Lisep lstm: a machine learning algorithm for early detection of septic shock. Sci. Rep. 9, 15132. 10.1038/s41598-019-51219-4 31641162 PMC6805937

[B9] Giannini H. M. Ginestra J. C. Chivers C. Draugelis M. Hanish A. Schweickert W. D. (2019). A machine learning algorithm to predict severe sepsis and septic shock: development, implementation and impact on clinical practice. Crit. care Med. 47, 1485–1492. 10.1097/CCM.0000000000003891 31389839 PMC8635476

[B10] Hancock J. T. Khoshgoftaar T. M. (2020). Catboost for big data: an interdisciplinary review. J. big data 7, 94–45. 10.1186/s40537-020-00369-8 33169094 PMC7610170

[B11] He H. Bai Y. Garcia E. A. Li S. (2008). Adasyn: adaptive synthetic sampling approach for imbalanced learning. In 2008 IEEE international joint conference on neural networks IEEE world congress on computational intelligence Ieee, 1322–1328.

[B12] Henry K. E. Hager D. N. Pronovost P. J. Saria S. (2015). A targeted real-time early warning score (trewscore) for septic shock. Sci. Transl. Med. 7, 299ra122. 10.1126/scitranslmed.aab3719 26246167

[B13] Hotchkiss R. S. Moldawer L. L. Opal S. M. Reinhart K. Turnbull I. R. Vincent J.-L. (2016). Sepsis and septic shock. Nat. Rev. Dis. Prim. 2, 1–21. 10.1038/nrdp.2016.45 PMC5538252 28117397

[B14] Hyland S. L. Faltys M. Hüser M. Lyu X. Gumbsch T. Esteban C. (2020). Early prediction of circulatory failure in the intensive care unit using machine learning. Nat. Med. 26, 364–373. 10.1038/s41591-020-0789-4 32152583

[B15] Ke G. Meng Q. Finley T. Wang T. Chen W. Ma W. (2017). Lightgbm: a highly efficient gradient boosting decision tree. Adv. neural Inf. Process. Syst. 30.

[B16] Khoshnevisan F. Chi M. (2020). “An adversarial domain separation framework for septic shock early prediction across ehr systems,” in 2020 IEEE international conference on big data (big data) (IEEE), 64–73.

[B17] Khoshnevisan F. Ivy J. Capan M. Arnold R. Huddleston J. Chi M. (2018). “Recent temporal pattern mining for septic shock early prediction,” in 2018 IEEE international conference on healthcare informatics (ICHI) (IEEE), 229–240.

[B18] Kim J. Chang H. Kim D. Jang D.-H. Park I. Kim K. (2020). Machine learning for prediction of septic shock at initial triage in emergency department. J. Crit. care 55, 163–170. 10.1016/j.jcrc.2019.09.024 31734491

[B19] Kumar A. Roberts D. Wood K. E. Light B. Parrillo J. E. Sharma S. (2006). Duration of hypotension before initiation of effective antimicrobial therapy is the critical determinant of survival in human septic shock. Crit. care Med. 34, 1589–1596. 10.1097/01.CCM.0000217961.75225.E9 16625125

[B20] Lewis R. J. (2000) “An introduction to classification and regression tree (cart) analysis,” in *Annual meeting of the society for academic emergency medicine in San Francisco, California* (Citeseer), 14.

[B21] Lin C. Zhang Y. Ivy J. Capan M. Arnold R. Huddleston J. M. (2018). “Early diagnosis and prediction of sepsis shock by combining static and dynamic information using convolutional-lstm,” in *2018 IEEE international conference on healthcare informatics (ICHI)* (IEEE), 219–228.

[B22] Liu R. Greenstein J. L. Granite S. J. Fackler J. C. Bembea M. M. Sarma S. V. (2019). Data-driven discovery of a novel sepsis pre-shock state predicts impending septic shock in the icu. Sci. Rep. 9, 6145. 10.1038/s41598-019-42637-5 30992534 PMC6467982

[B23] Misra D. Avula V. Wolk D. M. Farag H. A. Li J. Mehta Y. B. (2021). Early detection of septic shock onset using interpretable machine learners. J. Clin. Med. 10, 301. 10.3390/jcm10020301 33467539 PMC7830968

[B24] Mollura M. Romano S. Mantoan G. Lehman L.-w. Barbieri R. (2020). “Prediction of septic shock onset in icu by instantaneous monitoring of vital signs,” in 2020 42nd annual international conference of the IEEE engineering in medicine and biology society (EMBC) (IEEE), 2768–2771. 10.1109/EMBC44109.2020.9176276 33018580

[B25] Muralitharan S. Nelson W. Di S. McGillion M. Devereaux P. Barr N. G. (2021). Machine learning–based early warning systems for clinical deterioration: systematic scoping review. J. Med. Internet Res. 23, e25187. 10.2196/25187 33538696 PMC7892287

[B26] Singer M. Deutschman C. S. Seymour C. W. Shankar-Hari M. Annane D. Bauer M. (2016). The third international consensus definitions for sepsis and septic shock (sepsis-3). Jama 315, 801–810. 10.1001/jama.2016.0287 26903338 PMC4968574

[B27] Tomašev N. Glorot X. Rae J. W. Zielinski M. Askham H. Saraiva A. (2019). A clinically applicable approach to continuous prediction of future acute kidney injury. Nature 572, 116–119. 10.1038/s41586-019-1390-1 31367026 PMC6722431

[B28] Wardi G. Carlile M. Holder A. Shashikumar S. Hayden S. R. Nemati S. (2021). Predicting progression to septic shock in the emergency department using an externally generalizable machine-learning algorithm. Ann. Emerg. Med. 77, 395–406. 10.1016/j.annemergmed.2020.11.007 33455840 PMC8554871

[B29] Webb G. I. Keogh E. Miikkulainen R. (2010). Naïve bayes. Encycl. Mach. Learn. 15, 713–714. 10.1007/978-0-387-30164-8_576

[B30] Wright R. E. (1995). Logistic regression.

[B31] Yee C. R. Narain N. R. Akmaev V. R. Vemulapalli V. (2019). A data-driven approach to predicting septic shock in the intensive care unit. Biomed. Inf. insights 11, 1178222619885147, 10.1177/1178222619885147 PMC6829643 31700248

