# Supplementary material for: Development of continuous warning system for timely prediction of septic shock
Source: Front Physiol. 2024 Nov 20;15:1389693. doi: 10.3389/fphys.2024.1389693 (PMC11614766; doi:10.3389/fphys.2024.1389693)
Supplement: Supplementary file 1 [file Table1.pdf]

Table 1. Summary of previous early warning systems developed for septic shock

Author(Year)	Continuous Warning	Timely Range	Performance
?[1]	Capable but evaluated as screening system	Whole time range before the onset of target event	AUROC: 0.83, sensitivity: 0.85, specificity: 0.67, median lead time: 28.2 hrs
?	Incapable	Within 12 hours after admission	AUROC: 0.9411, F1 score: 0.8623, accuracy: 0.8658, recall: 0.8408, precision: 0.8849
?	Incapable	Before 3 hours from the onset of event	AUROC: 0.8647, F1 score: 0.7731, accuracy: 0.7747, recall: 0.7676, precision: 0.7931
?	Incapable	Within 8 hours after admission	AUROC: 0.895, F1 score: 0.808, accuracy: 0.813, recall: 0.787, precision: 0.830, Dataset: EHR from Christiana Care Health System
?	Incapable	Before 4 hours from the onset of event	AUROC: 0.943, F1 score: 0.868, accuracy: 0.875, recall: 0.826, precision: 0.915
?[1]	Incapable	Before 20 hours from the onset of event	Accuracy: 0.8312, sensitivity: 0.7812, specificity: 0.8663
?	Capable but evaluated as screening system	Whole time range before the onset of target event	Sensitivity: 0.26, specificity: 0.98, PPV: 0.29, NPV: 0.97, median lead time: 5hr 25min
?[1]	Capable but evaluated as screening system	Whole time range before the onset of target event	AUROC: 0.93, sensitivity: 0.88, specificity: 0.84, precision: 0.52, median early warning time: 7 hrs
?[1]	Capable but evaluated as screening system	Whole time range before the onset of target event	AUROC: 0.93, median hours before onset: 28.2 hrs
?[1]	Incapable	Before 24 hours from the onset of the event	AUROC: 0.81, sensitivity: 0.79, specificity: 0.66, PPV: 0.46, NPV: 0.90
?	Incapable	Before 48 hours from the onset of event	AUROC: 0.793, F1 score: 0.737, accuracy: 0.741, recall: 0.732, precision: 0.737
?[2]	Incapable	At the start of ED admission (warning based on triage information)	AUROC: 0.902, AUPRC: 0.556, sensitivity: 0.706, specificity: 0.900, PPV: 0.427, NPV: 0.967
?[1]	Incapable	Before 15 minutes from the onset of event	AUROC: 0.93, F1 score: 0.84, accuracy: 0.85, sensitivity: 0.89, specificity: 0.82, PPV: 0.80, NPV: 0.90
?	Incapable	Within 6 hours after admission	AUROC: 0.9483, sensitivity: 0.8392, specificity: 0.8814
?[3]	Capable but evaluated as screening system	Before 8 hours from the onset of the event	AUROC: 0.8, sensitivity: 0.85, specificity: 0.67
?[4]	Incapable	Before 4 hours from the onset of event	AUROC: 0.9087, accuracy: 0.8312, recall: 0.7812, precision: 0.8039, specificity: 0.8663

[1] The datasets used in these systems were from the MIMIC-II or MIMIC-III databases. While the MIMIC-IV dataset may share some common cohorts with these earlier versions, the EHR system schematics were significantly updated in MIMIC-IV, making direct comparisons between the methods of each study and our method challenging.

[2] All performances are those from ensemble(averaging) with baseline predictors only where the target event was the onset of septic shock within 20 hours after admission.

[3] Some performances are reported just with lower bound, and specificity is reported only with a graphic, necessitating approximation.

[4] All performances are those from logistic regression with E1 experiment result.

Table 2. Predictive performances of TEW3S in evaluation window –8 to 0

Evaluation Metric	All Shock	First Shock
TER	0.9403	0.9314
TAR	0.2018	0.1784
TER Stay	0.9314[1]	0.9347
TAR Stay	0.4305	0.7717

1 TER stay of all shock prediction always equals to TER of the first shock prediction.

Table 3. TER variation in various evaluation windows

Evaluation Window	-8 to 0	-8 to -1	-8 to -2
TER	0.9403	0.8230	0.7537
Evaluation Window	-7 to 0	-7 to -1	-7 to -2
TER	0.9382	0.8166	0.7452
Evaluation Window	-6 to 0	-6 to -1	-6 to -2
TER	0.9307	0.8049	0.7324
Evaluation Window	-5 to 0	-5 to -1	-5 to -2
TER	0.9254	0.7953	0.7175
Evaluation Window	-4 to 0	-4 to -1	-4 to -2
TER	0.9168	0.7836	0.6962

Table 4. Clinical variable level comparison between false negative cases and false positive cases

Variables	False Negative	False Positive
MAP (Mean Arterial Pressure, mmhg)	76.75	76.41
Lactate(mmol/l)	1.36	2.42
Arterial pH	7.40	7.37
GCS (Glasgow Coma Scale)	9.98	9.33
Creatinine(mg/dL)	1.56	1.80
Bilirubin(mg/dL)	2.28	3.54
Platelets(K/uL)	217.67	194.41
SOFA (Sequential Organ Failure Assessment)	7.29	8.01

Table 5. Misalignment proportions of conventional metrics

Type of Metric	Metric	Proportion of Discrepancy	Max TER	Max TAR
Cohort Based	AUROC	1	0.92	0.21
Cohort Based	F1-Score	0.91	0.92	0.21
Time Point Based	AUPRC	0.70	0.91	0.21
Time Point Based	F1-Score	0.70	0.91	0.21
